# An international comparison of longitudinal health data collected on long COVID in nine high income countries: a qualitative data analysis

**DOI:** 10.1186/s12961-025-01298-9

**Published:** 2025-03-24

**Authors:** Josephine Exley, Edmund Stubbs, Raphael Wittenberg, Nicholas Mays

**Affiliations:** 1 NIHR Policy Innovation and Evaluation Research Unit (PIRU), Department of Health Services Research and Policy, London School of Hygiene & Tropical Medicine, London, UK; 2 NIHR Policy Innovation and Evaluation Research Unit (PIRU), Care Policy and Evaluation Centre, London School of Economics and Political Science, London, UK

**Keywords:** Long COVID, Post COVID-19 syndrome, Health monitoring systems, Health research data and infrastructure, Longitudinal health data, Health registries

## Abstract

**Background:**

Long coronavirus disease (COVID) presents a significant health challenge. Long-term monitoring is critical to support understanding of the condition, service planning and evaluation. We sought to identify and examine longitudinal health data collected on long COVID to inform potential decisions in England regarding the rationale for data collection, the data collected, the sources from which data were collected and the methods used for collection.

**Methods:**

We included datasets in high-income countries that experienced similar coronavirus disease 2019 (COVID-19) waves to England pre-vaccine rollout. Relevant datasets were identified through literature searches, the authors’ networks and participants’ recommendations. We undertook semi-structured interviews with individuals involved in the development and running of the datasets. We held a focus group discussion with representatives of three long COVID patient organisations to capture the perspective of those with long COVID. Emergent findings were tested in a workshop with country interviewees.

**Results:**

We analysed 17 datasets from nine countries (Belgium, Canada, Germany, Italy, the Netherlands, New Zealand, Sweden, Switzerland and the United Kingdom). Datasets sampled different populations, used different data collection tools and measured different outcomes, reflecting different priorities. Most data collection was research (rather than health care system)-funded and time-limited. For datasets linked to specialist services, there was uncertainty surrounding how long these would continue. Definitions of long COVID varied. Patient representatives’ favoured self-identification, given challenges in accessing care and receiving a diagnosis; New Zealand’s long COVID registry was the only example identified using this approach. Post-exertion malaise, identified by patients as a critical outcome, was absent from all datasets. The lack of patient-reported outcome measures (PROMs) was highlighted as a limitation of datasets reliant on routine health data, although some had developed mechanisms to extend data collection using patient surveys.

**Conclusions:**

Addressing research questions related to the management of long COVID requires diverse data sources that capture different populations with long COVID over the long-term. No country examined has developed a comprehensive long-term data system for long COVID, and, in many settings, data collection is ending leaving a gap. There is no obvious model for England or other countries to follow, assuming there remains sufficient policy interest in establishing a long-term long COVID patient registry.

**Supplementary Information:**

The online version contains supplementary material available at 10.1186/s12961-025-01298-9.

## Background

Long coronavirus disease (COVID) is a major legacy issue of the coronavirus disease 2019 (COVID-19) pandemic [1–8]. The World Health Organization estimates that 17 million people in Europe alone were experiencing long COVID symptoms in 2022 [7]. The term long COVID was created by patients early on in the pandemic in the absence of an internationally agreed definition of the condition [9–11]. Long COVID is also commonly referred to as post COVID-19 syndrome, post COVID-19 condition, long-haul COVID-19, chronic COVID-19 and post-acute sequelae of SARS-CoV-2 (PASC) in the literature [12]. The National Institute for Health and Care Excellence (NICE) in the United Kingdom identifies long COVID as a multi-system condition with a range of debilitating symptoms that continue or develop after acute COVID-19 and are not explained by an alternative diagnosis [13]. It includes both ongoing symptomatic COVID-19 (from 4 to 12 weeks) and post-COVID-19 syndrome (12 weeks or more). Uncertainty persists around the proportion who will fully recover [14].

Research has identified more than 200 symptoms with impacts on multiple organ systems [15]. Long COVID is having a significant long-term impact on the health, wellbeing, daily activities and ability to work of those experiencing prolonged symptoms, as well as wider effects on their families and society [6, 14, 16–24]. Beyond the devastating personal impacts, the prevalence and complex nature of the condition pose considerable implications for both the health and care systems, as well as to the economy [18, 20, 22, 23]. In the United Kingdom, a survey by the Trade Union Congress found that 20% of people with long COVID were on sick leave and 16% were working reduced hours [25], while another study estimated 0.3% of the total working population have left employment owing to long COVID [26].

Long-term monitoring and follow up of people with long COVID is therefore critical to support our understanding of the condition, including the prevalence, natural history and long term outcomes, and to understand which treatments, interventions and care models work best and for whom. Data are also needed to support service improvement to understand demand for services, equity of access and potential areas of unmet need, and to support service planning, delivery and evaluation [3, 27]. To support this endeavour there is a need to develop or adapt existing data systems to identify and follow individuals with long COVID over time. Recommendations have been made in a number of countries to establish a long COVID disease register. For example, the Bevan Commission in Wales recommended the Welsh Government take steps to consolidate existing databases and resources held within different organisations and recruit a self-referred population of people with long COVID [28, 29].

In this study, we sought to identify and examine longitudinal health data collected on long COVID across high income countries to inform potential decisions on developing a long COVID data set for England by identifying:  the rationale for data collection, the data collected, the sources from which data were collected and the methods used for collection.

## Methods

### Selection of long COVID datasets

We selected higher income countries, with well-developed health and social care data systems, that experienced similar COVID waves to England pre-vaccine rollout. These countries were Austria, Belgium, Canada, England, France, Germany, Italy, the Netherlands, Spain, Sweden and Switzerland. To identify potentially relevant long COVID datasets, we searched PubMed for research outputs and commentaries, Google for news articles, and relevant government department and public health bodies’ websites within each country, as well as the authors’ professional networks and the recommendations of country experts and individuals approached to participate. Searches varied between platforms. We used broad search terms such as “long COVID” and “data collection” to capture any ongoing research about long COVID. We ran the searches in the languages of the countries we were finding datasets in, but only reviewed English language publications. We reviewed any sources which referenced collecting or utilizing existing data on long COVID, to determine how data were collected and from which datasets. We undertook follow-up searches of the identified long COVID data collection activities to obtain more information and manually reviewed reference lists of identified journal papers and reports to find other relevant sources. Relevant long COVID datasets included any dataset which captured individual-level data related to long COVID and reported repeat information on the included participants. We attempted to obtain the contact details of staff or authors to invite for interview.

On advice from experts, we extended our list of countries to include New Zealand, despite it not having experienced similar COVID waves pre-vaccination, on the grounds that it was distinctive in having established a national registry.

### Data collection

We conducted semi-structured interviews with informants involved in the development, management, collection and analysis of the identified datasets. They were purposively selected on the basis of their close involvement in the development and/or running of these datasets and had sufficient familiarity with the datasets to be able to respond to our questions. Further participants were identified through the authors’ networks and on the basis of recommendations from individuals who had already been approached to participate.

Potential informants were invited by email and provided with background information on the purpose of the study. Interviews were conducted on the video conferencing platform Zoom between 10 October 2022 and 9 February 2023. All interviews were conducted in English and, with consent, were audio-recorded, lasting between 1 and 1.5 h. One interview was not audio-recorded; instead, notes were taken.

The research team designed the topic guide (Appendix 1). Questions covered the objectives of data collection; how long COVID is defined and how participants are sampled and recruited or identified within existing data; how (and for how long) individuals are followed-up; the measures collected across the different levels of the health and social care system, demographic data, as well as service use and the outcomes captured; management and governance of the data; funding sources; and perception of data quality.

NHS England commissioned our work as a rapid scoping exercise, so we did not have time to consult patient groups about topic guide questions. However, to capture the patient perspective on the utility of the existing data and implications for future data collection in England, we undertook a focus group discussion with representatives of three long COVID patient groups, one United Kingdom-based (Long COVID Support United Kingdom) one German (Long COVID Deutschland) and one pan-European (Long COVID Europe). A summary of early findings was shared in advance of the discussion. The discussion focused on three questions: (1) what should be the aims of collecting data related to long COIVD; (2) who should be included in a long COVID dataset; and (3) which measures should be collected? The discussion took place over Zoom.

### Analysis

Transcripts and audio recordings of the interviews with country informants were reviewed by two team members, J.E. and E.S. Data were extracted by the same team members into a common extraction table to capture information on the history and development of each dataset, its objectives and accomplishments, sampling and recruitment methods, the measures collected, management and governance of data, funding, support from other organisations and general views from informants on the quality of the data. Information from literature and any additional documentation shared by informants was also added to the extraction table. J.E. or E.S. independently populated the extraction tables for half of the interviews and reviewed each others’ entries against interview transcripts, highlighting points of disagreement or missing information. The populated coding frame was shared with interviewees via email for their review and to clarify any points of uncertainty that had arisen during the coding. Extraction tables and subsequent drafts of the analysis were shared with senior team members, N.M. and R.W., for input and feedback.

### Developing recommendations

All participants in the study, including patient group members, were invited to attend an online workshop on 23 May 2023. The purpose of the workshop was to share the key study findings and reflect on the future direction of data collection related to long COVID. Through breakout discussions, participants were asked to consider why data should be collected on long COVID, what data should (or should not) be collected and how such data should be collected. Breakout rooms were used to enable attendees to debate these three points. Time was also allocated for all attendees to share and discuss each breakout room’s insights. The online workshop was recorded with the permission of attendees and notes were taken by the research team to capture the breakout discussions. The workshop both corroborated the research team’s initial findings and recommendations, and generated additional insights that informed the development of the study recommendations.

### Ethics

The project was approved by the London School of Hygiene & Tropical Medicine’s research ethics committee, approval number: 28096. All informants gave written consent to take part in the study.

## Results

We conducted interviews with individuals representing 17 longitudinal datasets from nine countries (Table 1 and Appendix 2). In Spain, individuals from the Spanish Network for Research on Long COVID (REiCOP) and the Spanish Society of General and Family Doctors (SEMG) informed us they were in the preliminary stages of developing a register (REGICOVID-AP Clinical Registry), but within the timeframe of this study we were not able to collect further details [30]. We were not able to identify any examples of longitudinal data collection in Austria or France.

**Table 1:** Overview of the aims of data collection, when data collection started and ended

Country	Dataset	Stated aims	Start date	End date
Group 1				
Canada	Canadian COVID-19 Antibody and Health Survey (CCAHS) [31, 32]	To evaluate the extent of the health status associated with the COVID-19 pandemic such as active COVID-19 infections and the prevalence of COVID-19 antibodies among a representative sample of Canadians. The survey also provides a platform to explore emerging public health issues, including the impact of COVID-19 on health and social wellbeing To determine the prevalence of post-COVID symptoms in the country: 1. Which symptoms occur 2. Assess whether such individuals are using the healthcare system 3. To understand pre-existing conditions and nonassociated conditions	April 2022	June 2023
Switzerland	Zurich Vaccine Cohort (part of the Swiss Immunitas Research Program) [33]	To investigate the immune response to COVID vaccines licensed in Switzerland in the Zurich population	April 2021	April 2024
United Kingdom	Office for National Statistics COVID-19 Infection survey (ONS-CIS) [34–36]	To estimate the extent of transmission and ongoing rates of infection in the United Kingdom	April 2020	March 2023
Group 2				
Belgium	COVIMPACT cohort study [37, 38]	To assess, among people with a recent COVID-19 infection, the evolution of their physical, mental and social health over the medium (min 3 months) and long term (max 2 years) and the factors associated with an (un)favourable evolution 1. Assess the type and duration of post-COVID condition (PCC) symptoms over time (that is, between 3 months and 2 years after infection) and in the different follow-up periods (that is, according to different COVID-19 variants and seasons) 2. Assess the determinants of short (min 3 months) and long term (max 2 years) PCC symptoms such as underlying chronic conditions, severity of the infection, hospitalisation, socioeconomic status, vaccination status, or health behaviours 3. Assess the short and long term evolution of (a) quality of life, (b) mental health, (c) functional limitations and (d) social contacts and employment among people with PCC compared with those without persistent COVID-19 symptom and with a similar sociodemographic sample of the general population not infected with COVID-19	April 2021	April 2023
Germany	German National Pandemic Cohort Network (NAPKON) [39–42]	To establish a standardized, high-quality data and biosample collection on patients, citizens and controls with comparator respiratory infections 1. Investigate frequency, severity and distinct phenotypes of COVID-19 and post-COVID-19 syndrome (PCS) in the German population and identify long-term clinical trajectories of PCS 2. Identify genomic, epigenomic, transcriptomic, proteomic and metabolomic signatures predicting course and outcome of acute and post-acute COVID-19 3. Decipher further central pathophysiologic mechanisms of specific COVID-19 related pathologies to inform development of therapies 4. Establish commonalities and differences between COVID-19 and other forms of respiratory tract infections, pneumonia and acute respiratory distress syndrome (ARDS) in detail 5. Understand reasons for development of acute or post-acute COVID-19 in SARS-CoV-2 vaccinated patients	July 2020	Nov 2022
Netherlands	RIVM Long COVID study [43, 44]	To assess whether the prevalence of fatigue, myalgia and other symptoms after COVID-19 exceeds the background prevalence in the general population, as well as prevalence in people with acute symptoms who tested negative for SARS-CoV-2. Further, to assess whether it is possible to predict which individuals will go on to experience persistent symptoms	Spring 2021	Spring 2023
Switzerland	Zurich Coronavirus Cohort (part of the Swiss Immunitas Research Program) [45, 46]	To investigate the long-term clinical outcomes, immune responses, transmission pathways and viral genetics in a representative, population-based cohort of persons infected with the coronavirus and their close contacts in the Canton of Zurich	August 2020	August 2023
Group 3				
Italy	Analysis and Response Strategies for the Long-Term Effects of COVID-19 Infection (Long-CoViD CCM) [47, 48]	Aims developed by the national project. Working on the first objective of the national project to estimate the cost of COVID-19 infection to the health service: 1. To indirectly assess the impact of long COVID by comparing healthcare utilization rates of exposed (previous SARS-CoV-2 infection) and unexposed (uninfected) 2. To assess utilization by care setting and epidemic phase of acute infection 3. “The aim of this project is to collect, retrospectively, data about patients who were positive […] then they became negative […] comparing to other patients that were negative […] to see what happens after a COVID patient became negative. In terms of hospital admissions, in terms of drugs administered, laboratory exams and diagnostic imaging and the related costs of these”	February 2020	December 2021
Netherlands	NIVEL Combined Primary Care Dataset (PCD) & Persistent Complaints after COVID-19 Project [49–51]	To characterize long COVID symptoms including providing insight into the nature, severity and duration of complaints, the risk factors for and underlying causes of long COVID and to map out the so-called care pathways of individuals with long COVID	April 2021	April 2022
Sweden	SCIFI-PEARL (Swedish Covid-19 Investigation for Future Insights—a Population Epidemiology Approach using Register Linkage) project [52, 53]		A large-scale effort to assemble a rich database for the study of Covid-19 epidemiologically in real time, i.e., with the requirement that data would be regularly and sufficiently frequently updated. The importance of not only focusing on COVID-19 patients was also recognized, making the inclusion of a large comparison cohort reflecting the general population another key asset. 1. To descriptively characterize individuals with COVID-19 (overall, hospitalized, intensive care unit (ICU) treated, deaths and in important subgroups) in terms of sociodemographics, various risk factors (for example, comorbidity and co-medication), geographic distribution and timing, among others. Further, to describe how the epidemic and the characteristics of the affected patient group change over time, that is, the so-called natural history of the epidemic in relation to the population. 2. To quantify the impact and importance of comorbidities (in particular, respiratory and cardiovascular, diabetes and obesity), severity of and co-medications for concomitant disease, sociodemographics and other risk factors, as well as protective measures and vaccinations, on risk of various endpoints. Some anticipated endpoints include developing COVID-19, being hospitalized for COVID-19, emergency/ICU care, treatments, specific care, hospitalization duration, prognosis, death and in survivors, long-term outcomes up to 3 years. Various study designs and methods as appropriate will be employed, including case-cohort, case-control and interrupted time series study designs and machine learning methods. 3. To develop and periodically update prediction models for COVID-19 occurrence and prognosis and evaluate how this impacts healthcare resource use, including the need for ICU treatment. Use of machine learning methods will be important here. Secondary aims include:1. Treatment effects of medicines and other healthcare interventions, including novel indications for established treatments and vaccinations and possible side effects related to COVID-19. 2. Resource use in the healthcare system, at the individual level and at societal level (hospital resources, measures, medicines and costs, among others) 3. Study the effectiveness of vaccination programs and possible adverse reactions in the population. 4. Consequences of the COVID-19 pandemic including future vaccination programs on drug use, health care and other societal functions in the population. 5. Other upcoming questions and information gaps from society, healthcare and the scientific community (if these are outside the above specified issues, may need additional supplemental ethical application)	2025
United Kingdom	CVD-COVID-UK/COVID-IMPACT [54–58]	CVD-COVID-UK aims to understand the relationship between COVID-19 and cardiovascular diseases such as heart attack, heart failure, stroke and blood clots in the lungs. COVID-IMPACT expands the approach to examine the impact of COVID-19 on other health conditions and their related risk factors	October 2020	Ongoing: data collection covered by control of patient information (COPI) notices
Group 4				
Belgium	Post-COVID pathway [59]	To have a quality indicator of the guideline that has been developed, the number of patients and whether they receive care in accordance with the guideline	July 2022	Ongoing: individuals enrolled in the care pathway, funded by social health insurance
Canada	Post-COVID-19 Interdisciplinary Clinical Care Network (PC-ICCN) [60–63]	To identify, implement and evaluate clinical care programs and health policy initiatives that improve the care of patients with persistent symptoms post COVID‐19 using a learning health system approach Aim to use the data for long term research and to tweak services/advocate for different services. At the heart of their objectives is improving care offered to long COVID patients and to “provide services relevant to needs”: (a) to demonstrate the ability to stand up interdisciplinary clinics offering care and education to patients suffering from long COVID, with standardized follow up and data capture (b) to acquire the expertise and experience to deliver high quality interdisciplinary care (c) to develop accessible tools for primary care physicians and patients to enable capacity building with that acquired expertise and (d) to use data acquired in this context to inform the evolution of care models The secondary goals were to normalize the embedding of research into clinical care and to create a culture of collaboration across different clinical and research teams	July 2020	Ongoing: tied to clinics, which shifted online in spring 2023
Germany	Outpatient treatment of COVID-19 infections (ABC19 study) [64]	To collect long-term and systematic data from the outpatient care of COVID-19 patients and to better understand the course of the disease in COVID-19 patients. In particular, examining the influence of comorbidities on the course of COVID-19	March 2020	No defined endpoint
Italy	National surveillance on the long-COVID condition	To define the number and characteristics of centres assisting patients with long COVID manifestations in Italy	February 2022	May 2022
England	NHSE Long COVID registry [65–68]	To understand the longitudinal patient journey and support operational, clinical and research activities and allow a more granular understanding of healthcare access for groups who experience health inequalities	July 2021	Ongoing: tied to clinics. Individuals followed until discharged from clinics
Group 5				
Aotearoa New Zealand	Long COVID Registry Aotearoa New Zealand [69, 70]	To estimate the clinical, quality of life and economic impacts of long COVID and provide a means to continually monitor health outcomes and inequities	July 2023	Ongoing: surveys follow-up for 6 months, beyond which followed in EHRs

The included datasets sampled different populations, used different data collection tools and measured different outcomes. We grouped the datasets into five types on the basis of the population sampled and the data collection tools used: (1) population surveys (*n* = 3); (2) surveys of individuals with a positive COVID-19 test (*n* = 4); (3) individuals with a COVID-19 test result identified in routine health data (*n* = 4); (4) datasets of individuals who had sought health care specifically for long COVID (*n* = 5) and (5) individuals who self-reported or self-identified as having long COVID (*n* = 1). Datasets were managed by a mixture of local and national governmental departments, academic institutions and independent nonprofit foundations (working with the healthcare sector).

Most of the datasets examined in this study were time-limited. Dataset types 1, 2 and 3 (see Table 1) had either ended or were winding down. Likewise, specialist long COVID services in all countries we examined were at that moment only seeing patients for a limited period – patients in British Columbia attended post-COVID-19 recovery clinics for up to 18 months, after which care reverted to their GPs, while the Belgian care pathway covered care for up to 12 months. In all countries, there was uncertainty over how long these specialist services would continue to be funded. The Aotearoa New Zealand long COVID register was conducting surveys for 6 months post-recruitment, beyond which participants were to be followed up in electronic health records (EHRs).

### Why collect data on long COVID?

The purpose of data collection varied across the different types of datasets, providing insight into different research and policy priorities (Table 1). Type 1 and 2 datasets aimed to establish the population prevalence of COVID-19 and long COVID, the risk factors for developing long COVID, disease development and prognosis and the impacts of long COVID on individuals. The Belgian COVIMPACT, Dutch RIVM Long COVID, the German NAPKON, the Swiss Immunitas Research Program and the United Kingdom ONS-CIS were set up before long COVID had been formally characterized, and, as such, long COVID was not the primary research focus these surveys sought to address [33, 35]. In contrast, the Canadian CAHS was established specifically to determine the prevalence of persistent COVID-19 symptoms, contact with and use of the health system and the relationship with pre-existing conditions [31]. Like the Canadian CAHS, the Aotearoa New Zealand long COVID registry (type 5) was developed specifically to address research questions related to long COVID, to estimate the clinical, quality of life and economic impacts of long COVID in New Zealand, as well as continually monitoring health outcomes and inequities [71].

Like type 2 datasets, those in type 3 also examined the long-term impacts of having had a COVID-19 infection. Both the Italian Long-CoViD CCM and the Swedish SCIFI-PEARL examined the impact of COVID-19 infection on the health care system, while the Dutch NIVEL combined primary care dataset (PCD) and persistent complaints aimed to map out the so-called care pathways of individuals with long COVID [47, 48, 50, 52, 53, 72].

Type 4 datasets were specifically focused on individuals accessing specialist services to understand the care they received and (in some cases) inform the development of care models [48, 60, 61]. Finally, several datasets aimed to examine the relationship between COVID-19 and pre-existing conditions, including the Canadian CAHS, the United Kingdom CVD-COVID-UK and the German ABC19 study [31, 56, 58, 64].

Interviewees across countries discussed the need for longer-term patient monitoring and follow-up to improve the understanding of long COVID. Interviewees and workshop participants commented that the heterogeneity in purpose across datasets reflected the limited understanding of the condition and its impacts. They identified multiple data needs including to support understanding of the prevalence, risk factors and progression of the condition, patients’ journeys and use of health care, treatment effectiveness and the study of the personal impact on those living with long COVID.

### Who is data collected from?

An overview of the population included, and the recruitment methods, is presented in Table 2.

**Table 2:** Overview of who is included in data collection

Dataset	Definition of long COVID	Recruitment	Number of participants	Control group
Group 1				
Canada/CCAHS	Symptoms: experiencing symptoms three or more months after initial COVID-19 symptoms/positive test/thought had COVID-19	Stratified sample representative of adults at the provincial and national level aged 18+ years	Approximately 32 500	• Includes individuals with and without COVID-19 infection • Includes individuals self-reporting experiencing and not experiencing long COVID symptoms
Switzerland/Zurich Vaccine Cohort	Diagnosis: having received a diagnosis from a clinician; Self-identify: participants self-identifying as having long COVID and Symptoms: based on WHO and NICE recommendations, self-reporting still experiencing symptoms more than 3 months after infection	Age stratified random sample of adults 18+ years old attending vaccine centre for their first vaccine in Zurich	575	• Includes individuals with and without COVID-19 infection • Includes individuals self-reporting experiencing and not experiencing long COVID symptoms • Individuals with COVID-19 infection from study E7
United Kingdom/ONS CIS	Symptoms: experiencing symptoms more than 4 weeks after they first had COVID-19 (not reliant on positive test result), that are not explained by something else (based on NICE definition)	Individuals aged 2+ years, randomly selected from households in the United Kingdom where a member had previously participated in an ONS or NISRA survey	Approximately 21 000 individuals from 10 000 households	• Includes individuals with and without COVID-19 infection • Includes individuals self-reporting experiencing and not experiencing long COVID symptoms
Group 2				
Belgium/COVIMPACT	Diagnosis: having received a diagnosis from a clinician and Symptoms: in line with NICE guidelines, long COVID defined as having symptoms related to COVID-19 more than 3 months after the acute infection	Adults aged 18+ years living in Belgium who received a positive PCR test at a national testing centre were sent an invitation to the baseline survey when contacted by the test and trace team	10 708	• Individuals that recovered and did not experience long COVID symptoms • Matched control from the Belgian Health Interview Survey on the basis of age, sex and level of education
Germany/NAPKON	Symptoms: persistent symptoms after testing positive for COVID	Cross-sectional platform (SUEP): individuals of any age attending inpatient and outpatient settings with confirmed acute SARS-CoV-2 infection. All university and non-university hospitals and primary care practices can become study sites. Some mobile study teams cover long-term care and rehabilitation facilities High-Resolution Platform (HAP): adults (≥ 18 years) with SARS-CoV-2 hospitalized at one of the 10 participating university hospitals Population-Based Platform (POP): adults (≥ 18 years) living in one of the three study regions who had a positive test recorded and contacted by local health authority at least 6 months before the start of the study. Individuals with acute re-infection at the time of the interview or scheduled site visit are excluded Participating study sites consecutively enrol all eligible patients. The recruitment for each cohort is balanced for disease severity. Additional stratification criteria for age, sex, infection date or vaccination status apply depending on the cohort	7093 total: SUEP: 2749 HAP: 710 POP: 3634 There is no overlap between the cohorts	Comparator respiratory infections
Netherlands/RIVM long COVID	Symptoms: experiencing symptoms for at least 2 months, which are significantly elevated compared with controls	Prospective cohort: individuals (aged 5+ years) who received a positive test for COVID at a national test and trace centre invited to participate when contacted by the municipal health service informing them of their test results. Had to complete the baseline questionnaire within 7 days of the positive test Retrospective cohort: individuals (aged 5+ years) who tested positive for COVID at a national test centre and DID NOT complete the baseline questionnaire within 7 days. Individuals who self-identify as having long COVID and had COVID at any time since the start of the pandemic (confirmed by test or unconfirmed) could self-enrol	Study includes 50 000 participants total Prospective cohort: 8000–9000	• Negative COVID test result • Matched control from the Basic Registration of Persons without a positive test for SARS-CoV-2 infection or known history of probable infections
Switzerland/Zurich coronavirus cohort	Diagnosis: having received a diagnosis from a clinician; Self-identify: participants self-identifying as having long COVID; and Symptoms: based on WHO and NICE recommendations, self-reporting still experiencing symptoms more than 3 months after infection	Age-stratified random sample of adults aged 18+ years who had a positive lab test reported to the local public health authorities in the Canton of Zurich All close contacts were tested and a selection of close contacts that tested negative were tested again at the end of quarantine period. Close contacts that tested positive invited to participate in the study as index cases	1552	Individuals that recovered and did not experience long COVID symptoms
Group 3				
Italy/long-CoViD CCM	No formal definition of long COVID; the study is examining the impact of the post COVID phase of the disease by following individuals beyond the acute infection period	Adults aged 40+ years in regional administrative database with a COVID-19 laboratory test results recorded	600 000 individuals (300 000 positive test; 300 000 negative test)	Matched control negative test result
Netherlands/NIVEL-PCD	Symptoms: COVID-19 confirmed by laboratory test in GP data (or during the first wave suspected by algorithm) and experiencing symptoms 3–12 months after infection that were not present or less severe before COVID-19 infection Strict definition: at least two symptoms recorded in International Classification of Diseases (ICD) codes judged to be related to COVID-19 or post COVID by experts (one of which must be a so-called core symptom – a symptom/complaint identified on the basis of comparison with controls or WHO definition) 3–12 months after infection and visited a GP more than two times for these symptoms Broader definition: at least two symptoms recorded in ICD codes judged to be related to COVID-19 or post COVID by experts (one of which must be a so-called core symptom – a symptom/complaint identified on the basis of comparison with controls or WHO definition) 3–12 months after infection. Does not include GP visits as anecdotal evidence suggesting that a number of individuals with post COVID are seeking care from other sources and did not want to miss this group	EHR: All patients who seek care at a GP in the NIVEL network Corona cohort: individuals with a positive COVID infection (either from municipal health service or before community testing set-up, identified on the basis of symptoms in patient record) automatically flagged in GP records	EHR: Approx. 1.7 million individuals from 500 GP practices Corona cohort: 442 patients with COVID infection from 25 GP practices	• EHR: matched control negative test result • EHR: before and after COVID pandemic • Corona cohort: includes individuals self-reporting experiencing and not experiencing long COVID symptoms
Sweden/SCFI-PEARL	Diagnosis/clinical codes: Initially used UO-9, ICD-10 code Symptoms: symptom profiles recorded in EHR	All individuals with a positive PCR test result identified from SmiNet (the national register of notifiable communicable diseases managed by the Public Health Agency of Sweden)	Whole Swedish population	Random sample of general population from the national register of the Total Population at Statistics Sweden
United Kingdom/CVD-COVID-UK/COVID-IMPACT	Diagnosis/clinical codes: SNOMED-CT codes in primary care data and ICD-10 codes in hospital data	All patients in routine health datasets	England: 57 million Scotland: 5.5 million Wales: 3.2 million	Feasible to construct controls within dataset
Group 4				
Belgium/Post COVID pathway	Clinical assessment: still suffering from symptoms related to COVID-19 (fatigue, shortness of breath and cognitive dysfunction, among others) having a visible impact on their daily life 12 weeks after first symptoms of COVID-19 infection and/or 12 weeks after a positive COVID-19 test	All patients in GP records who had entered the long COVID Care pathway	Unknown	None
Canada/PC-ICCN	Clinical assessment: based on GP’s assessment. Eligibility based on WHO’s definition of having had confirmed COVID-19 infection either hospitalized or managed as outpatients and experiencing symptoms for 12 weeks or more that could not be explained by an alternative diagnosis	All adults aged 18+ years referred by a primary care provider to one of five post-COVID recovery clinics	October 2022: 7649 clinic visits with 3241 patients 6176 questionnaires completed by 3366 patients	None
Germany/ABC19	Clinical assessment: GPs are provided with a list of symptoms in a public catalogue to guide their clinical decision making, but study has not provided any clear criteria to define a long COVID patient	Any GP offices can sign-up to participate GPs recruited patients aged 14+ years attending with acute COVID infection. Amended to include any patients who had previously had a confirmed COVID infection and were seeking care for COVID related symptoms	400 patients from 16 GP offices	None
Italy/National surveillance	Not defined. Recruited any individual attending any service for long COVID on the basis of self-definition	Recruited any clinic or centre providing services to people with long COVID. Includes both single specialty sites and multidisciplinary sites, as well as those that were set up specifically to treat long COVID and those that were not. Does not include primary care Participating facility required to recruit at least 20 patients (12 +) per month. Centres with a higher case volume could choose to select an index week in the month and recruit all patients in that week if they wished	137 centres from 17 Italian regions and two autonomous provinces registered on the online platform. Of these, 124 (90.5%) from 16 regions and two autonomous provinces completed the online questionnaire	None
England/NHSE long COVID registry	Clinical assessment: GP or other clinician can refer patient with ongoing symptoms lasting 4 weeks or more following COVID illness	All patients attending a post COVID service for an initial assessment or triage	99 adult and 10 children services July 2023; 113 453 individuals referred	None
Group 5				
Aotearoa New Zealand/long COVID registry	Self-identify: based on experiencing symptoms for 12 weeks or more after acute infection. People who are unsure whether symptoms are long COVID can completed a quiz at longcovidsupport.co.nz	Adults aged 18+ years living in New Zealand with self-reported long COVID	Aiming to recruit 3000 (1348 as of March 2024)	Possibility to undertake some matching in routine health data

### Defining long COVID

The emergent nature of the condition and a lack of a clear definition was identified as a key challenge among interviewees. The definition used varied across datasets and was based on symptoms, diagnosis, clinical assessment or self-identification (see Table 2). Most commonly, long COVID was defined in line with the WHO’s definition, on the basis of individuals’ self-reported experience of symptoms beyond the acute stage of illness, although the time point post-infection varied. Datasets that drew on routine data relied on diagnostic codes. However, several interviewees discussed concerns about the reliability and validity of diagnostic codes, in particular, doubts about clinicians’ familiarity with the codes, whether they were using them routinely and applying them uniformly. Patient representatives also expressed concerns that diagnostic codes are not accurately capturing individuals with long COVID.

“Set definition is UO-8 code. But it was a new code, and nobody knew how to use it, so I think there’s been both overuse and underuse in different areas and in different parts of the health care system” (university researcher, Sweden, SCIFI-PEARL).

An explicit aim of the NIVEL-PCD in the Netherlands and the Swedish Covid-19 Investigation for Future Insights – a Population Epidemiology Approach using Register Linkage (SCIFI-PEARL) was to characterize long COVID using different definitions [49, 52, 73].

### Population captured

The population captured also varied across datasets. Type 1 datasets recruited a representative sample of the general population irrespective of participants’ COVID-19 or long COVID status. Type 2 datasets sampled individuals who had tested positive for COVID-19; in general, individuals were recruited when they received their test results from their national or regional testing programme. In most contexts, research access to testing data had been made available under special legislative powers to tackle COVID-19. A Swiss interviewee commented that:

“[T]his setup is far from common in Switzerland, so it’s really rare to establish such a collaboration between the research group and a governmental body because of all the privacy issues, etcetera” (university researcher and physician, Switzerland, Zurich Coronavirus Cohort).

Type 3 datasets tracked all individuals with a positive COVID-19 test or a long COVID diagnosis in national or regional EHRs. Like type 2 datasets, the respondent from the Dutch NIVEL-PCD reported that access to testing data in medical records was only made available under special legislation, and access was granted for a period of about one year. The Italian Long COVID dataset intended to collect data from all individuals with a positive test result in regional administrative data, but, during acute phases of the pandemic, the Department of Prevention “encountered considerable difficulties in monitoring all the positives over time” (senior director, National Institute of Health).

The five type 4 datasets captured individuals who had sought health care specifically for long COVID. The examples from Canada, England and Italy captured individuals who had been referred to specialist services, while those in Belgium and Germany included individuals receiving care in primary care settings. Patient representatives voiced concerns that only examining individuals accessing services (type 3 and 4) would fail to capture a representative sample of individuals living with long COVID, given the challenges that individuals with long COVID have experienced in accessing care and receiving a diagnosis. Workshop participants also highlighted the inherent biases in collecting data only from individuals accessing specialist clinics and the impact this is likely to have on understanding of the epidemiology. They told us that data collected from individuals accessing specialist services are not likely to be generalizable beyond those settings. For example, the Canadian PC-ICCN interviewee commented on the inequitable access to the clinic, noting the “higher rates of hospitalization with COVID among non-Caucasian individuals, but those in post-recovery clinics are mainly Caucasian”(manager, Provincial Health Research Services Authority).

Patient representatives’ preferred method of recruitment was self-referral, as used by the Aotearoa New Zealand long COVID registry (type 5), which allows any individual self-identifying with long COVID to sign up online. However, some interviewees voiced concerns related to the representativeness of data where individuals self-refer. Only 8.4% of participants in the Aotearoa New Zealand registry are Māori (compared with 19.6% of the general population), hence active recruitment in Māori and Pasifika communities to increase registration among these groups [74]. The lack of representation of minoritized ethnic groups and those from lower socioeconomic groups was also noted among the other dataset types. For example, the German NAPKON study received almost no non-German consent forms, suggesting the non-German speaking population are underrepresented, while the Dutch Long COVID study only captured “5 to 6 per cent of people with migration background whereas population-wide this proportion is very different” (interview with two non-university researchers, National Institute for Public Health).

### Control group

To characterize the condition and identify risk factors associated with developing long COVID, the interviewees highlighted the importance of including a comparison group given that many of the long COVID symptoms reported are nonspecific and prevalent in the general population. Only type 1, 2 and 3 datasets included a control group. The interviewee from New Zealand told us that whilst there is no control group “linkage with the IDI [Integrated Data Infrastructure, a large research database that holds data about life events, such as education, income, benefits, migration, justice and health] will allow for us to undertake some matching”. Control groups differed by data source and comprised individuals with and without self-reported symptoms, matched controls from those who had never tested positive for COVID-19 or who had other respiratory illnesses and random samples of the general population. Datasets using EHRs were also able to compare against pre-pandemic trends.

### Which data were collected and how?

#### Survey versus use of routine health records

Type 1, 2 and 5 datasets collected primary data using self-reported surveys. In addition, the Dutch NIVEL-PCD (type 3) and the Canadian PC-ICCN (type 4) undertook patient surveys for a sub-set of individuals and type 4 datasets included surveys completed with a clinician. A reported advantage of surveys over EHRs was the flexibility to add questions and the ability to capture the fluctuating and episodic nature of the condition. These attributes were cited as particularly valuable in capturing long COVID considering that the condition and its impacts have been poorly described.

“[W]hen we set up the study, we did not know that long COVID is going to be a thing. We only wanted to track health status over time. [...] The studies did prove to be a very flexible tool in a way, and we were able to adapt questionnaires to emerging questions, add questions that may have been important” (university researcher and physician, Switzerland, Corona Immunitas Research Programme).

Another reported advantage of surveys over EHRs was the ability to collect patient-reported outcome measures (PROMs).

“When it comes to policy making these impact on your life questions, if you can work, what is the quality of your life, you don’t really get from a registry, an electronic health record, these are all very important” (interview with two university researchers, Netherlands, Long COVID study).

“I think the best data is coming from the patient side. Maybe this is, we should build it, shift the focus more on the patient side, so that the workload is a little bit more on the patient’s side and less on the physician side, and we try to simplify our CRF” (manager, Germany, ABC19).

#### Outcome measures

The most important outcome to assess, according to the patient representatives, was post-exertion malaise (PEM). This is one of 12 core outcomes that researchers have recommended should be evaluated in all research studies and in clinical care for people with long COVID [79–81]. PEM was not included in any of the datasets examined. Table 3 presents the included measures mapped against the PC-COS (an international consensus study developing a standardized set of outcomes for people with long COVID) and demonstrates the heterogeneity between datasets in terms of both the outcomes measured and the measurement instruments used. The most frequently collected outcomes relate to the impact of symptoms on daily life, health-related quality of life, respiratory functioning and mental health.

**Table 3:** Included studies mapped against the core outcome set for adults with post-COVID-19 condition [79, 81]

Core outcome	Measurement instrument	Group 1 (*n* = 3)	Group 2 (*n* = 4)	Group 3 (*n* = 3)	Group 4 (*n* = 5)	Group 5 (*n* = 1)
Physiological or clinical outcomes						
1. Cardiovascular functioning, symptoms and conditions	Circulation subscale of the Symptom Burden Questionnaire for Long COVID (SBQ-LC) New York Heart Association (NYHA) Functional Class					
2. Fatigue or exhaustion	Fatigue Assessment Scale (FAS) Fatigue Severity Scale (FSS) Functional Assessment of Chronic Illness Therapy – Fatigue (FACIT-F)	• Switzerland_ Zurich Vaccine Cohort (FAS)	• Belgium_COVIMPACT (Visual Analogue Scale for Fatigue) • Germany_NAPKON (Chalder Fatigue Scale, FACIT-F) • Netherlands_RIVM Long COVID (CIS) • Switzerland_1 (FAS)		• Canada_ PC-ICCN (FSS) • Italy_1 (6-min walking)	• Aotearoa New Zealand_Long COVID registry (FAS)
3. Pain	Brief Pain Inventory (BPI)		• Germany_NAPKON (DN2, HIT-6)			• Aotearoa New Zealand_Long COVID registry (BPI-SF)
4. Nervous system functioning, symptoms and conditions	Central Sensitization Inventory (CSI)					
5. Cognitive functioning, symptoms and conditions	Cognitive Failures Questionnaire (CFQ) Montreal Cognitive Assessment ‘Blind’ version (MoCA-Blind)		• Germany_ NAPKON (PROMIS Kognition) • Netherlands_ RIVM Long COVID (CFQ)		• Canada_ PC-ICCN (neurological screen) • Italy_ National surveillance (MoCA)	
6. Mental functioning, symptoms and conditions	Generalized Anxiety Disorder 7 (GAD-7) PTSD Checklist for DSM5 (PCL-5)	• Switerzerland_ Zurich Vaccine Cohort (DASS21)	• Belgium_ COVIMPACT (GAD-7, PHQ-9, Visual Analogue Scale for Life Satisfaction) • Germany_ NAPKON (GAD_7, Brief Resilience Scale) • Netherlands_ RIVM Long COVID (HADS) • Switerzerland_ Zurich Coronavirus Cohort (DASS21)		• Canada_ PC-ICCN (GAD-7, PCL-5) • Italy_ National surveillance (HADS)	• Aotearoa New Zealand_Long COVID registry (GAD-7, PHQ-9, Kessler Psychological Distress Scale [K10])
7. Respiratory functioning, symptoms and conditions^a^	mMRC Dyspnoea Scale	• Switzerland_ Zurich Vaccine Cohort (mMRC)	• Belgium_ COVIMPACT (mMRC) • Germany_ NAPKON (mMRC, PROMIS Dyspnoea) • Netherlands_ RIVM Long COVID (shortness of breath) • Switzerland_ Zurich Coronavirus Cohort (mMRC)	• Netherlands_NIVEL (shortness of breath and influence of shortness of breath on daily life)	• Canada_PC-ICCN (cough visual analogue scale and UCSD-SOBQ) • Italy_National surveillance (mMRC and diffuse capacity for carbon monoxide)	• Aotearoa New Zealand_ Long COVID registry (mMRC)
8. Post-exertion symptoms	De Paul Symptom Questionnaire (DSQ-PEM)					
Life impact outcomes						
9. Physical functioning, symptoms and conditions	“Impact on Daily Life” of the Symptom Burden Questionnaire for Long COVID (SBQ-LC)	• Canada_CCAHS (whether symptoms related to COVID-19 limit daily activities) • UK_ONS-CIS (whether symptoms reduce ability to carry out day-to-day activities compared with before they had COVID-19) • Switzerland_ Zurich Vaccine Cohort (impact of symptoms on daily activities and consider self back to pre-infection health status)	• Belgium_ COVIMPACT (Washington City Group (WCG) and Global Activity Limitation Indicator (GALI)) • Germany_ NAPKON (activities of daily living, Barthel index and Clinical Frailty Scale) • Netherlands_ RIVM Long COVID (IPAQ, impact complaints have had on life, control over life, worry about complaints and impact on mood) • Switzerland_ Zurich Coronavirus Cohort (impact of symptoms on daily activities and consider self back to pre-infection health status)	• Netherlands_NIVEL (hours spent seated and participation in activities of moderate to vigorous intensity)	• Canada_PC-ICCN (PHQ-2) • Germany_ABC19 (impact of symptoms on daily life)	• Aotearoa New Zealand_Long COVID registry (C19-YRS)
10. Work or occupational and study changes	Work Ability Index questionnaire (WAI) Work Productivity and Activity Impairment (WPAI) questionnaire “Your day-to-day work/school?” question item from WHO Global COVID-19 Clinical Platform Case Report Form for Post COVID condition	• Canada_CCAHS (impact of work/school and employment history) • UK_ONS-CIS (current work/education status)	• Belgium_ COVIMPACT (change in employment and income)	• Netherlands_NIVEL (absence from work, impact of COVID on working hours and change in working activities)		• Aotearoa New Zealand_Long COVID registry (work and social adjustment scale [WSAS])
Survival						
11. Survival^a^	Time until death			• Sweden_SCIFI-PEARL (cause of death)		
Outcome from previous COS						
12. Recovery^a^	Recovery Scale for COVID-19					
Other PROMs measured not in COS						
Health-related quality of life		• Switzerland_ Zurich Vaccine Cohort (EQ-5D)	• Belgium_ COVIMPACT (EQ-5D) • Germany_2 (EQ-5D) • Netherlands_ RIVM Long COVID (EQ-5D, RAND-12/SF-12, RAND-36) • Switzerland_1 (EQ-5D)	• Netherlands_NIVEL (SF-12) • Sweden_SCIFI-PEARL (RAND-36)	• Canada_PC-ICCN (EQ-5D) • Germany_ABC19 (EQ-5D) • Italy_National surveillance (EQ-5D)	• Aotearoa New Zealand_Long COVID registry (EQ-5D)
Odour and taste			• Netherlands_ RIVM Long COVID (odour and taste capabilities)			
Substance abuse					• Canada_PC-ICCN (CAGE Alcohol Questionnaire)	
Social health			• Belgium_ COVIMPACT (UCLA Three-Item Loneliness Scale)			• Aotearoa New Zealand_Long COVID registry (LCSS)

^a^Only outcomes for which consensus was agreed on the measurement instrument

*C19-YRS* COVID-19 Yorkshire Rehabilitation Scale, *CIS* Checklist Individual Strength, *DASS21* Depression Anxiety Stress Scale-21, *IPAQ* International Physical Activity Questionnaire, *LCSS* Long Covid Stigma Scale, *PHQ* Patient Health Questionnaire, *PROMIS* Patient-Reported Outcomes Measurement Information System, *SF-12* Short Form 12, *UCSD-SOBQ* University of California, San Diego Shortness of Breath Questionnaire

The lack of PROMs was a notable gap in those datasets that rely solely on EHRs (type 3 datasets and Belgium Post-COVID care pathway and NHS England Long COVID registry). The Dutch NIVEL-PCD (type 3) has captured PROMs by making use of software previously developed to automatically flag patients with a COVID-19 diagnosis (see Panel 1) [51, 75]. The dataset includes EHRs for everyone attending primary care and extensive PROMs on a subset of individuals who tested positive for COVID-19, including services not captured in the EHR such as mental health care received, self-care and data on quality of life, lifestyle and employment. At present, PROMs are not routinely collected across services by the English NHS Long COVID registry, although there is ongoing work to enable the reporting of EQ-5D-5L [68, 76]. Additionally, a digital platform completed on a smartphone web application has been developed to collect PROMs in 40 post-COVID clinics across the country, although the PROMs collected varies between clinics [77, 78].

Panel 1 NIVEL research primary care data set and corona cohort

**Table:** 

The NIVEL Primary Care Database (PCD) and Corona Cohort in the Netherlands is an example that allows the long-term follow up of patients in electronic health records (EHRs), supplemented by the patients’ perspective through a short-term study. The NIVEL-PCD includes the EHRs of roughly 1.7 million individuals who have sought care at one of the 500 GP practices inside the NIVEL network. NIVEL’s data platform links EHR records from general practitioners, out-of-hours general practitioners and hospitals. The database is updated annually for all practices and weekly for 350 practices. The team at NIVEL had previously developed infrastructure to automatically flag individuals within the database with particular characteristics, which they were able to use early in the pandemic to identify individuals who had had COVID-19 [51, 75]. The EHR of participating practices (*n* = 18) was scanned to flag individuals aged 16 years and older with a positive COVID-19 result. Following review of identified patients by their GP, all eligible patients were invited by post to participate in the “persistent complaints after COVID-19 project”. Consent was sought to participate in surveys at baseline and after 3, 6 and 12 months and to link their survey data to the PCD. In total, 442 respondents participated in the NIVEL Corona Cohort, of whom 421 (95%) consented to have their survey data linked to their EHR.

### Burden of data collection

While surveys were reported to offer flexibility, issues were raised in relation to the burden that they can place on both patients and clinicians. For example, the survey used by the Italian National surveillance system was developed from the WHO’s Global COVID-19 Clinical Platform Case Report Form (CRF) for Post-COVID Condition [82], but it was considered overly burdensome for clinicians to complete. The process of refining the survey was reported by the interviewee to be challenging given the lack of consensus on many aspects of long COVID.

"It was very debated what to collect because clinicians had very different opinions [...] some wanted a lot of data collected, others would focus on some core data. In the end, we collect three kinds of data. We collect symptoms as defined by the patients. Then we collect new diagnoses, which is what the physicians believe the patient had or have. And then we collect some, but not too many data on tests [...] we decided that, diving into laboratory tests was very complicated and we decided to simplify and not collect, for example, blood tests. We put more interest in collecting data on quality of life, on anxiety and depression and on diagnoses” (senior director, Italy, National surveillance).

The German NAPKON study (type 2) captured the largest volume of primary data of any of the datasets examined, with over 3000 data items collected [40], which the interviewee considers contributed to drop out. Likewise, the Belgium post-COVID care pathway and the German ABC-19 interviewees reported that there was a lot of pressure on primary care and GPs during the pandemic, and it was challenging for them to engage with data collection on top of their already heavy workload.

“When the registry was ready to go the second wave came in, and nobody had time to think about research. And the next thing was, when the wave was gone, we started to vaccinate people, the same doctors that they are focused on COVID had to vaccinate all people. So we had a lot of obstacles to get our registry running” (manager, Germany, ABC-19).

### Benefits of data linkage

Large scale data systems that link data from different parts of the health and care system have been developed in several countries (type 3). For example, the CVD-COVID-UK dataset and the Swedish-SCFI-PEARL datasets are highly comprehensive datasets that use individual identity numbers to link a diverse range of routine health data, including primary and secondary care data and existing disease registers. The Swedish SCIFI-PEARL links 20 different registers/EHRs to identify patients diagnosed with COVID-19 across Sweden. The database includes all individuals with a positive polymerase chain reaction (PCR) test identified in SmiNet (the national register of notifiable communicable diseases managed by the Public Health Agency of Sweden), as well as patients with relevant COVID-19 ICD-10 and procedure codes in routine health records and individuals whose cause of death was recorded as being due to COVID-19. Health care contacts for COVID-19 in primary care are only captured in two regions, which cover roughly 40% of the Swedish population [52]. The National Register of the Total Population from Statistics Sweden, a representative sample of the general population, has been used to construct different comparison groups as required for different statistical analyses. The CVD-COVID-UK/COVID-IMPACT Consortium includes 57 million patient records across England, 5.5 million across Scotland and 3.2 million across Wales. CVD-COVID-UK links primary care data, hospital episodes (covering inpatient, outpatient, emergency department and critical care episodes), registered deaths (including causes of death), COVID-19 laboratory tests, community dispensed medicines, specialist intensive care, cardiovascular audit, hospital electronic prescribing and COVID-19 vaccination data [54].

Access to data compiled in the CVD-COVID-UK/COVID-IMPACT dataset had been enabled under time-limited control of patient information (COPI) notices, issued by the Secretary of State for Health and Social Care to require organisations to share confidential patient information with approved users for COVID-19 research purposes without requiring patients’ consent. A challenge raised by the interviewee was that “until such time as there are equivalent datasets available under different provision notices, if we broaden our scope at the moment, we would lose access to certain datasets including primary care” (project manager/university researcher, HDR UK).

Likewise, some type 4 datasets have made use of linking to reduce the burden of primary data collection. For example, the Canadian PC-ICCN originally captured a larger number of diagnostic tests and extensive bloodwork, such as computed tomography, echocardiogram, pulmonary function tests and 6-min walking tests, but like the Italian example, these were dropped following an evaluation of their utility for clinical decision-making to reduce the burden of testing on patients and the health care system. Instead, patients were invited to participate in the provincial biobank network, which collects blood samples that can be linked to the registry. A total of four other datasets collected biosamples (two type 1 datasets [Canadian CHAS, UK ONS-CIS] and two type 2 datasets [German NAPKON, Zurich Coronavirus Cohort]). The Dutch NIVEL-PCD (type 3) had planned to collect biosamples, but because of resource constraints this was not possible.

“We only administered questionnaire data. Probably it would be very useful to also have some measurements like immunological parameters for stuff you could measure in the laboratory. We didn’t do that, also because of pragmatic reasons especially at the scale we are including people. There was really an infrastructure problem setting up a study during a pandemic where everyone is stretched to their limits, so this was the most pragmatic solution” (non-university researcher, Netherlands, NIVEL-PCD).

### Length of patient follow-up versus data completeness

Length of follow-up varied across datasets (see Appendix 2). Like recruitment, interviewees noted higher loss to follow-up among some population groups. For example, the Belgium COVIMPACT study found men and individuals with lower education levels were more likely to be lost to follow-up. Some datasets, such as the Aotearoa New Zealand long COVID registry, have requested participants’ consent for their data to be linked to Statistics NZ’s Integrated Data Infrastructure using their National Health Index number, to enable follow-up in routine data after surveys end. However, patient representatives expressed concerns that datasets that rely on routine health system data for long term follow-up could miss the episodic nature of long COVID, potentially mischaracterizing individuals who are self-managing as having recovered.

“You’ve got to be really careful to ensure that people aren’t knocked off the register because nothing’s been logged for a matter of maybe weeks” (patient representative).

Interviewees from the Italian National Surveillance and the German ABC-19 studies echoed these concerns. Follow-up was reliant on patients returning to the clinic (only relevant to the GP follow-up component of the study), regardless of whether they were still experiencing symptoms; they voiced concerns that this might have led to higher loss to follow-up among individuals who recover, feel able to self-manage or no longer wish to attend the service.

## Discussion

Long COVID is having a long-lasting impact on the health, wellbeing, daily activities and livelihoods of those experiencing prolonged symptoms, as well as their families [83]. Even on the basis of conservative estimates, the burden of illness represents a challenge to the health and care system and the economy [5, 84]. Many gaps in the understanding of long COVID exist, and interviewees in this current study identified multiple outstanding research questions [13]. To address these gaps requires long-term monitoring of individuals with long COVID using data from different sources. In this study we examined 17 examples of longitudinal long COVID health datasets established in nine countries. The examples identified ranged from population surveys which captured individuals’ symptoms and experience of long COVID to a national register of individuals self-identifying as having long COVID.

The long COVID datasets examined in this study highlight the heterogeneity of approaches taken between countries to data collection, using different definitions of long COVID, populations and controls, outcomes and outcome measures [4, 12, 85]. The heterogeneity between datasets likely reflects the emergent nature of long COVID, the diverse interests of researchers and funders, differences in aims and the data sources available, as well as the different time points at which they were developed. For example, several of the patient surveys were established before long COVID had been characterized and were adapted to include questions related to long COVID once the research need had emerged, while others were designed to specifically examine long COVID.

The patient surveys in datasets type 1 and 2, and those associated with the Dutch NIVEL-PCD (type 3), had either ended or were about to end, leaving a gap in many data systems. Patient surveys were reported to be key to answer questions related to the prevalence, risk factors and evolution of the condition, as well as examination of the impacts of long COVID on individuals and their families. Patient surveys were seen to be particularly important to measure outcomes not captured in routine data, in particular, PROMs, and to characterize the fluctuating and episodic nature of long COVID. The challenge is how to sustain patient surveys in the long-term given the short-term nature of most research funding. A recent study of disease registers in the United Kingdom identified lack of long-term funding as the key threat to their sustainability [86]. For these disease registers, charities associated with the disease play a central role in providing some continuity of funding and running registers.

Patient representatives raised concerns, echoed by interviewees, that datasets that included only individuals with a positive COVID-19 test result recorded in routine data (type 2 and 3) or captured only individuals in EHRs (type 3) or only individuals accessing specialist services (type 4) will not provide a representative sample of individuals with long COVID. Not all individuals with long COVID had been tested for SARS-CoV-2, particularly at the start of the pandemic when many countries stopped or reduced testing in the community or, as highlighted by one of the Italian interviewees, when surveillance systems failed to record all test results as they became overwhelmed during peaks in infection. Further, in many countries, testing data were only made available to researchers for a limited time.

Patients in several countries have faced challenges in receiving a diagnosis and accessing care [87–90]; 36–42% of individuals included in the Aotearoa New Zealand Long COVID registry had not received a clinical diagnosis [74]. The lack of a standardized definition of long COVID, no diagnostic tests and low level of awareness of the existence of ICD codes for long COVID were reported to have resulted in heterogeneity in the use of ICD codes and underreporting of the condition. An analysis of OpenSAFELY data in England and ten United Kingdom longitudinal studies found the use of diagnostic codes to be low compared with survey data based on self-reported long COVID [91, 92]. Questions therefore remain about the reliance on ICD codes for long COVID, which is likely to limit what can be done using EHRs at present.

For conditions that are poorly characterized and/or where patients do not always receive a clinical diagnosis, it can be hard to accurately capture and track the affected population. Registries for conditions other than long COVID have faced similar challenges. For example, the lack of a consistent approach to diagnosis and misclassification of myalgic encephalomyelitis/chronic fatigue syndrome (ME/CFS) has led to the under-reporting of cases and insufficient research, medical care and treatment [93]. Similarly, for complex regional pain syndrome (CRPS), there is currently no clinically recognized diagnostic test. The CRPS Network has developed a broad definition and collects information that allows participants to be divided into further subgroups on the basis of different definitions of the condition [94]. Such an approach would enable researchers to continue to examine the accuracy of diagnostic coding, as with the Swedish SCIFI-PEARL study and the Dutch NIVEL-PCD (see Table 2), and it reflects the preference of long COVID patient representatives whom we consulted. They questioned the accuracy of coding in patients’ health records given the complex presentation of symptoms and the reported challenges experienced in receiving a diagnosis [9, 95].

Self-referral was the patient representatives’ preferred method of recruitment, to ensure data are captured from individuals accessing all types of health services, plus those who have not accessed any health services. Similarly, the Bevan Commission in Wales and the Inquiry into Long COVID and Repeated COVID Infections in Australia recommended that any future long-COVID registry should include a self-referred population along with routine data sources to adjust for recruitment bias and promote equitable access [28, 29, 96]. The Aotearoa New Zealand registry is the only example included in this study that is recruiting participants in this way. Researchers at Martin Luther University Halle-Wittenberg, TU Munich and Otto von Guericke University Magdeburg in Germany have established a long COVID register, which anyone over 18 years of age with self-reported symptoms can join [97]. Participants complete a questionnaire every 6 months and, at present, the funding is open-ended.

The pandemic was reported to have served as a catalyst for data sharing. Special legislation was introduced in several countries to provide time-limited access to data and to expedite consent and data linkage processes to support research on COVID-19. This was reported by several interviewees to have facilitated research that would not otherwise have been possible or would have taken much longer to set up. There is a need to review existing data access, including understanding the benefits and whether there have been any serious data breaches or patient harm as a result of providing so-called emergency access, as these arrangements raise questions around whether data should be made more readily available routinely for research purposes, including for long COVID.

### Strengths and limitations

The strengths of this study are that we conducted thorough searches for long COVID data sets in higher income countries, conducted interviews with informants involved in the development or running of these data sets, held a focus group discussion with long COVID patient representatives on the emerging findings and held an online workshop with the study participants to test the draft recommendations.

Undertaking a thorough search of longitudinal studies within countries was challenging, as many of the datasets had not started producing published outputs that could be identified through bibliographic database searching. We relied heavily on snowball sampling, recruiting initial interviewees through the authors’ own networks and then via the recommendations of individuals approached to participate in the study. Given these challenges, we are likely to have missed examples from countries within the scope of this study. A recent mapping of long COVID surveillance systems across the EU identified many of the same datasets as this study, but additionally identified an example in Germany we did not pick up and the Spanish REGICOVID-AP Registry. In the time available for data collection, we were not able to obtain sufficient information to confidently characterize these two datasets for inclusion in the study [98]. Further, the search was deliberately limited to countries with a similar healthcare system to England and which experienced similar COVID waves prior to the vaccine rollout; New Zealand was added once we found out about its distinctive approach to long COVID data collection, even though its pandemic experience was very different from England’s. There may have been equally interesting examples from countries other than New Zealand that we were unaware of.

Of the datasets examined, not all of them have published protocols or made their data collection tools publicly available. As a result, the amount of information available on each dataset varied. Interviews aimed to provided additional insight and fill the gaps in information, providing a more comprehensive overview of the datasets examined but varied in the level of detail that interviewees could provide.

### Recommendations

This study was commissioned to inform the further development of data systems for long COVID in England. The recommendations are informed by the findings of our interviews and workshop and, while they relate to the English context, they are likely to be generalizable to other settings.

First, there is a need to decide which questions the dataset should address. Determining the research priorities and the outcomes to be measured will require consultation with health authorities, patient organisations, clinicians and the research community, and it should be codesigned with people with lived experience of long COVID to ensure data are relevant and useful to those who will be using the data or may be affected by the findings. For example, the James Lind Alliance is an expert in helping patients, carers and clinicians work together to prioritize evidence needs through its Priority Setting Partnerships (https://www.jla.nihr.ac.uk/about-priority-setting-partnerships).

Given the definitional issues and the fact many individuals with long COVID are not (routinely) in contact with health care services, datasets should take an inclusive approach to capture a broad population using different definitions such as self-report, positive COVID test, recorded diagnosis and others, with a variable to indicate the basis of the individual’s inclusion. Such an approach would provide a deeper understanding of how long COVID is being experienced and enable data users to select subpopulations to examine particular questions. As part of efforts to improve the validity and completeness of data being collected, it will be important to keep clinicians, especially GPs, up-to-date on the clinical diagnostic coding of long COVID.

Different objectives will require different measures to be collected. For example, if the goal of research is to assess the impact of long COVID on people’s lives, then collecting employment and income data will likely be extremely useful alongside quality-of-life measures; this could be done either by linking data to employment records (if possible) or by collecting measures directly from people with long COVID about how their work has been affected. Alternatively, if establishing the risk factors and comorbidities for long COVID were the primary objective, then longitudinal diagnostic, symptom and healthcare usage data would need to be extracted from patient records, augmented, if possible, from patient surveys.

Datasets should include a range of outcomes measures, in particular PROMs, to shed light on quality of life and ability to function day-to-day. Since there is evidence that long COVID has significantly impacted individuals’ ability to work, data systems should aim to look beyond the clinical and health impacts to include labour market outcomes. There is a need to appraise the most effective and efficient way to collect the data, maintain the data set and make data available to health care providers, researchers, patients and others with an interest.

There is a strong case to build a population-based cohort study to follow individuals over longer time periods than many of the datasets identified here. For any data collection to achieve its objectives, there is a need to ensure that it has adequate funding for an extended period [86]. As COVID-19 and long COVID become lower priorities for governments, alternative funders are likely to be needed. Exploring the possibility and suitability of greater collaboration with ME/CFS organisations, as has been done in Australia, which expanded its ME/CFS Registry to include individuals with long COVID in October 2023, could be one route to maximize what can be achieved [99, 100].

## Conclusions

Long COVID affects the health and quality of life of millions of people and represents a significant long-term health challenge. There is a demand for data to support a greater understanding of the natural history of the condition, the long-term effects on individuals with long COVID and the effectiveness of the range of treatments and services to support those living with long COVID. Addressing these needs will require a mix of data sources that capture different populations with long COVID over the longer term. None of the countries examined have implemented a comprehensive dataset for long COVID. Many of the datasets examined have only been funded in the short-term. As a result, there is no obvious model for England or other countries to follow, assuming there remains sufficient policy interest in establishing a long-term long COVID patient registry. Reliance on routine health care data alone would leave a gap in data important for understanding long COVID. It is important that the development of a longitudinal health data set on long COVID should be based on careful consideration of the priority questions to be addressed, the views of stakeholders, including people with lived experience, and the importance of sustainability of the data collection and management.

## Supplementary Information

Supplementary Material 1.

Supplementary Material 2.

## Data Availability

The data analysed during the current study are not publicly available as the data contain potentially sensitive participant information. Consent was only sought to share anonymous quotations specifically for the purposes of this study but are available from the corresponding author on reasonable request.

## Abbreviations

ABC-19: Outpatient treatment of Covid-19 infections
BPI-SF: Brief Pain Inventory-Short Form
C19-YRS: COVID-19 Yorkshire Rehabilitation Scale
CCAHS: Canadian COVID-19 Antibody and Health Survey
CFQ: Cognitive Failures Questionnaire
CIS: Checklist Individual Strength
CRF: Clinical Platform Case Report Form
CRPS: Complex regional pain syndrome
DASS21: Depression Anxiety Stress Scale-21
EHRs: Electronic health records
FACIT-F: Functional Assessment of Chronic Illness Therapy – Fatigue
FAS: Fatigue Assessment Scale
GAD-7: Generalized Anxiety Disorder 7
ICD: International Classification of Diseases
Long-CoViD CCM: Analysis and Response Strategies for the Long-Term Effects of COVID-19 Infection
LCSS: Long COVID Stigma Scale
ME/CFS: Myalgic encephalomyelitis/chronic fatigue syndrome
mMRC: MMRC Dyspnoea Scale
MoCA: Montreal Cognitive Assessment
NAPKON: German National Pandemic Cohort Network
NICE: National Institute for Health and Care Excellence
ONS-CIS: Office for National Statistics COVID-19 Infection survey
PCC: Post-COVID Condition
PCD: Combined primary care dataset
PC-COS: Post-COVID Condition Core Outcomes
PC-ICCN: Post COVID-19 Interdisciplinary Clinical Care Network
PEM: Post-exertion malaise
PHQ-2: Patient Health Questionnaire-2
PROMIS: Patient-Reported Outcomes Measurement Information System
PROMS: Patient-reported outcome measures
PCL-5: PTSD Checklist for DSM5
REiCOP: Spanish Network for Research on Long COVID
SCIFI-PEARL: Swedish Covid-19 Investigation for Future Insights – a Population Epidemiology Approach using Register Linkage
SEMG: Spanish Society of General and Family Doctors
SF-12: Short Form 12
UCSD-SOBQ: University of California, San Diego Shortness of Breath Questionnaire
