# Supplementary material for: An international comparison of longitudinal health data collected on long COVID in nine high income countries: a qualitative data analysis
Source: Health Res Policy Syst. 2025 Mar 24;23:37. doi: 10.1186/s12961-025-01298-9 (PMC11931811; doi:10.1186/s12961-025-01298-9)
Supplement: Supplementary file 1 — Supplementary Material 1. [file 12961_2025_1298_MOESM1_ESM.docx]

## Introduction:

1. How do you define post-COVID or long-COVID syndrome and how do you identify these patients?
2. What data collection is currently ongoing in *[country]* related to post-COVID?

## Measures collected:

1. Do you capture information on individuals with post-COVID across the different tiers of the health and care system?

### Primary care

1. What demographic and socioeconomic data is collected on individuals with post-COVID in primary care?
   1. Is this data collection undertaken specifically for individuals with post-COVID or do you draw on existing data sources?
   2. Who collects and inputs the data to the system?
2. What patient-level data on receipt or use of primary care services by people with post-COVID is collected?

*Prompt: initial assessments, referrals to other services or to self-management resources, consultations, rates of patients not attending appointments etc.*

1. Is this data collection specific to individuals with post-COVID or are you drawing on existing data sources?
2. What data is collected on consultations related to post-COVID?
3. What data is collected on referrals to other services for post-COVID?
4. Who collects and inputs the data to the system?
5. What outcome measures are collected on patients with post-COVID in primary care?
6. Is this data collection specific to individuals with post-COVID or are you drawing on existing data sources?
7. Who inputs the data supplied to the collection?
8. Are these linked to patient-level data? If so, how?
9. At what level is data collected?
10. Do you collect any COVID-specific data at the primary care level?
11. Do you collect data on individual’s COVID vaccinations?
12. Do you collect data on individual’s COVID testing?
13. Do you collect information on COVID reinfections?

### Secondary care

1. Do you collect any additional or different demographic and socioeconomic data in secondary care from those already discussed?

*IF YES*:

1. Is this data collection undertaken specifically for individuals with post-COVID or do you draw on existing data sources?

*Prompt: Are these collected nationally, regionally, research purposes only?*

1. Who collects and inputs the data to the system?

*Prompt:* *clinicians, the patients themselves, service administrators etc.*

1. What patient-level data on receipt or use of secondary care services by people with post-COVID is collected?
2. Is this data collection specific to individuals with post-COVID or are you drawing on existing data sources?
3. What data is collected on advice and information offered to individuals with post-COVID?
4. What data is collected on medical tests and diagnostic procedures for post-COVID?
5. What data is collected on referrals to other services?
6. Who collects and inputs the data to the system?
7. What data is collected on use of mental health services?
8. What outcome measures are collected on patients with post-COVID in secondary care?
9. Is this data collection specific to individuals with post-COVID or are you drawing on existing data sources?
10. Who inputs the data supplied to the collection?
11. Are these linked to patient-level data? If so, how?
12. Do you collect any COVID-specific data at secondary care level, in addition to those we have already discussed?

### Long-term care

1. Do you collect any additional or different demographic and socioeconomic data in secondary care from those already discussed?

*IF YES*:

1. Is this data collection undertaken specifically for individuals with post-COVID or do you draw on existing data sources?
2. Who collects and inputs the data to the system?
3. What patient-level data on receipt or use of long-term care services by people with post-COVID is collected?
   1. Is this data collection specific to individuals with post-COVID or are you drawing on existing data sources?
4. What outcome measures are collected on patients with post-COVID in long term care?
5. Are there other key measures being collected that we haven’t discussed?
6. Is it possible to identify individuals and link them across the different datasets discussed?

## Management and governance of data:

1. Who manages and governs the data collection related to post-COVID?
2. Does the same individual have responsibility for its linkage with other data sources, and its storage?
3. Who is able to access data related to post-COVID?
4. Are there any restrictions on who can access the data?
5. How is data accessed?
6. At what level is data accessed?

## Participants:

1. Is there a defined endpoint to data collection?
2. Are individuals removed for any other reason?
3. Can they be re-added if they relapse?
4. Do you know or have a sense what proportion of the total affected population the post-COVID data collection captures?
5. How do know this*?*
6. Are there any plans to try and expand the dataset to capture a wider pool of individuals affected by post-COVID?
7. If no, do you think that it would be important to do so?

## Objectives and accomplishments:

We’d like to understand a bit more on why the data is being collected and what has been achieved so far.

1. What are the strategic aims of the data collection?
2. What are the reasons for undertaking the data collection?
3. Does this vary by user?
4. Are the aims the same across the health and care system?
5. Do you think any existing services have been improved because of the data collection?
6. Which services have been improved? *Prompt: primary, secondary, long-term*
7. In what way have they been improved?

IF NOT

1. Do you think there is the potential to improve services based on the data being collected?
2. Have any new services or treatments been offered or are under development as a result of analysis or research using the data collection?

IF NOT

1. Are there aspirations to use the data for service planning in the future?
2. Has the register been used to provide evidence to seek or justify further resources for post-COVID services?
3. What data items were used to justify this?
4. Have you got evidence of improved patient outcomes resulting from analyses or research using the data collection?
5. What evidence have you got and how did you collect it?
6. Do you think the data collection has had any impact on health inequalities?
7. Have inequalities in health outcomes or access to services been reduced?
8. To your knowledge, has the data collection been used to provide evidence to justify further research into post-COVID?
9. Which data items were used to justify this?

## Funding:

1. How is the data collection currently funded?
2. Do you anticipate funding will be maintained for the foreseeable future?
3. Through the same source of funding?
4. Why/why not?

## Support:

1. Which organisations or individuals led the development of the post-COVID disease data collection?
2. Did any other organisations help/support set up the register/dataset?
3. Support recruitment?
4. Ongoing maintenance of the register/dataset?
5. Were people with post-COVID or groups representing them involved in the development of the data collection?
6. If so, how and at which stages?
7. Did you use examples/expertise from any other disease case registers to establish the data collection for post-COVID?
   1. If so, how did they help?
   2. Did you draw on questionnaires from other diseases/countries to develop the questionnaire?

## Perceptions on quality of the dataset

1. Do you think that the data collection is of sufficient coverage and quality?
2. What could be done to improve it?
